# Ominous Sign of Pneumatosis Intestinalis With Portal Venous Gas

**DOI:** 10.7759/cureus.35605

**Published:** 2023-02-28

**Authors:** Brett Miller, Sobaan Taj, Viraaj S Pannu, Kameron Tavakolian, Liz Joseph, Mohammad A Hossain

**Affiliations:** 1 Internal Medicine, Jersey Shore University Medical Center, Neptune City, USA; 2 Medicine, Hackensack Meridian School of Medicine, Nutley, USA

**Keywords:** pneumatosis cystoides intestinalis, small-bowel obstruction, portal venous gas, gastric pneumatosis, pneumatosis intestinalis

## Abstract

The presence of gas and free air in the extraluminal space of the intestines is known as pneumatosis intestinalis (PI). There are many different causes of this finding, including gastrointestinal, pulmonary, autoimmune, and many more. It is often difficult to differentiate the etiology and clinical importance of the radiographic evidence on pneumatosis intestinalis due to the unclear pathophysiology causing the disease. To complicate things further, the ominous sign of portal venous gas poses the question of whether surgical intervention is needed. We report two cases both with clinical and radiographic evidence of secondary pneumatosis intestinalis with an associated sinister finding of portal venous gas. The cases differ by urgent surgical intervention versus observation before surgery. In this case series, we emphasize the importance of recognizing the radiographic finding and stress the need for further research to standardize a plan of care, including indications for surgery. We encourage more cases like this to be reported to aid in diagnosing and treating this condition early on with the aim of improving the mortality associated with it.

## Introduction

The presence of gas and free air in the extraluminal space of the intestines is known as pneumatosis intestinalis (PI) [1]. The findings can be further categorized into benign pneumatosis intestinalis and life-threatening pneumatosis intestinalis [2]. The rare finding was first discovered by Du Vernoy during an autopsy specimen in the 1730s and later named pneumatosis cystoidies intestinalis (PCI) in 1825 [3]. There are many different causes of this finding, including gastrointestinal, pulmonary, autoimmune, and many more. It is often difficult to differentiate the etiology and clinical importance of the radiographic evidence on pneumatosis intestinalis due to the unclear pathophysiology causing the disease. We report two cases both with clinical and radiographic evidence of secondary pneumatosis intestinalis with an associated sinister finding of portal venous gas. The cases differ by urgent surgical intervention versus observation before surgery. In this case series, we emphasize the importance of recognizing the radiographic finding and stress the need for further research to standardize a plan of care, including indications for surgery. We encourage more cases like this to be reported to aid in diagnosing and treating this condition early on with the aim of improving the mortality associated with it.

## Case presentation

Case one

A 40-year-old male with no significant past medical history presented to the emergency with severe and diffuse abdominal pain ongoing for one day. The pain was associated with constipation, nausea, vomiting, and scrotal swelling with right testicular pain. He denied fever, chills, diaphoresis, chest pain, shortness of breath, and diarrhea. Vital signs on admission were all within normal limits. A physical exam showed tenderness to palpation of the lower abdomen and a non-reducible right inguinal hernia with associated scrotal swelling. Labs were significant for white blood cell count (WBC) of 17.1 (4.5-11*10^3/uL), as mentioned in Table 1. An ultrasound of the scrotum showed right scrotal sac bowel loops consistent with inguinal hernia and no evidence of testicular torsion. An x-ray of the abdomen was also performed and showed multiple dilated loops of small bowel with multiple air-fluid levels compatible with bowel obstruction, but no free air was noted. The patient was admitted for incarcerated and possible strangulated right inguinal hernia with small bowel obstruction for which general surgery was consulted. 

**Table 1:** Laboratory results on admission

Test	Case 1	Case 2	Reference range
Complete blood count (CBC)			
White blood cells	17.1 10*3/µL	22.5 10*3/µL	(4.5 - 11.0 (10*3/µL)
Hemoglobin	15.5	12	(12.0 - 16.0 g/dL)
Hematocrit	44.9	40.7	(40 - 53%)
Platelets	240	260	(140 - 450*10^3/uL)
Complete metabolic panel (CMP)			
Blood urea nitrogen (BUN)	16	34	(5-25 mg/dL)
Creatinine	1.01	1.86	(0.62 - 1.24 mg/dL)
Sodium	136	132	(136 - 145mmol/L)
Potassium	4.1	5.4	(3.5 - 5.2mmol/L)
Chloride	99	93	(96-110mmol/L)
Carbon dioxide	22	22	(24-31mmol/L)
Anion gap	15	17	(5-13mmol/L)
Calcium	10.0	9.4	(8.5-10.5mmol/L)
Aspartate aminotransferase (AST)	26	21	(10-42u/L)
Alanine aminotransferase (ALT)	20	15	(10-60u/L)
Other labs			
Lactic acid	-	6.2	(0.5-2.0 mmol/L)
Imaging			
Presence of portal venous gas	Present	Present	Absent

**Figure 1:**
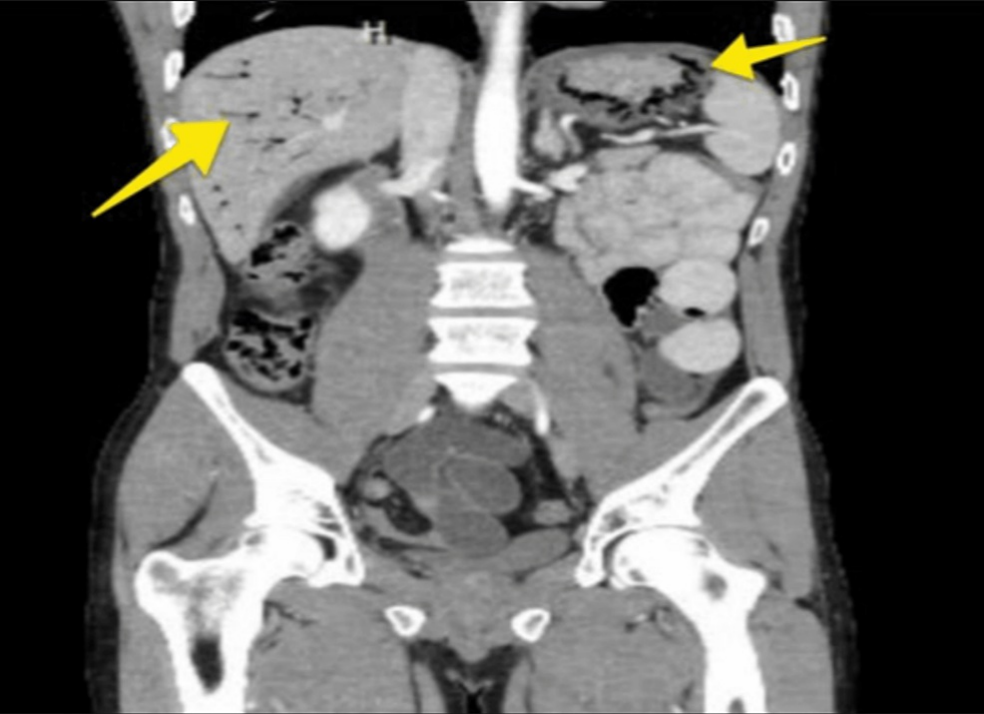
Coronal view of CT scan of the abdomen and pelvis with contrast showing gastric pneumatosis (right arrow), proximal duodenum pneumatosis, and portal venous gas (left arrow)

The patient was evaluated by general surgery with a recommendation for a computed tomography (CT) scan of the abdomen and pelvis with contrast before surgery. A CT scan of the abdomen confirmed small bowel obstruction secondary to right inguinal hernia. Additionally, the CT scan showed gastric and proximal small bowel pneumatosis, and portal venous gas (Figure 1). The incidental finding of pneumatosis intestinalis increased the concern for bowel ischemia. He was taken to the operating room with a preoperative diagnosis of incarcerated and possibly strangulated right inguinal hernia with small bowel obstruction, pneumatosis, and portal venous air. The patient underwent a laparoscopic right inguinal hernia repair with mesh placement by transabdominal pre-peritoneal technique and excision of cord lipoma. There was no evidence of strangulation. The patient tolerated the surgery well without any complications. After surgery, his diet was progressively advanced. He was cleared for discharge two days after surgery, with pain well controlled and tolerating a full diet. 

Case two

This is a 58-year-old male with a past medical history significant for hypertension, hyperlipidemia, alcoholism, umbilical hernia, peripheral neuropathy, and cerebral vascular accident two years prior with residual left-sided weakness who presented to the hospital with multiple diarrheal episodes and abdominal pain for one day. The patient woke up one night prior needing to go to the bathroom and had a diarrheal episode that was described as "dark" by the patient. The patient also reported having right and left lower quadrant abdominal pain that he described as dull, 10/10, constant, non-radiating, aggravated by movement with no alleviating factor. Associated symptoms include diaphoresis and shortness of breath without a cough. As per the patient, he had never experienced these symptoms in the past. The patient denied any recent travel, nausea, vomiting, fevers, chills, hematochezia, or hematemesis. The patient's last meal was take-out hot dogs, which he ate the night before he presented.

**Figure 2:**
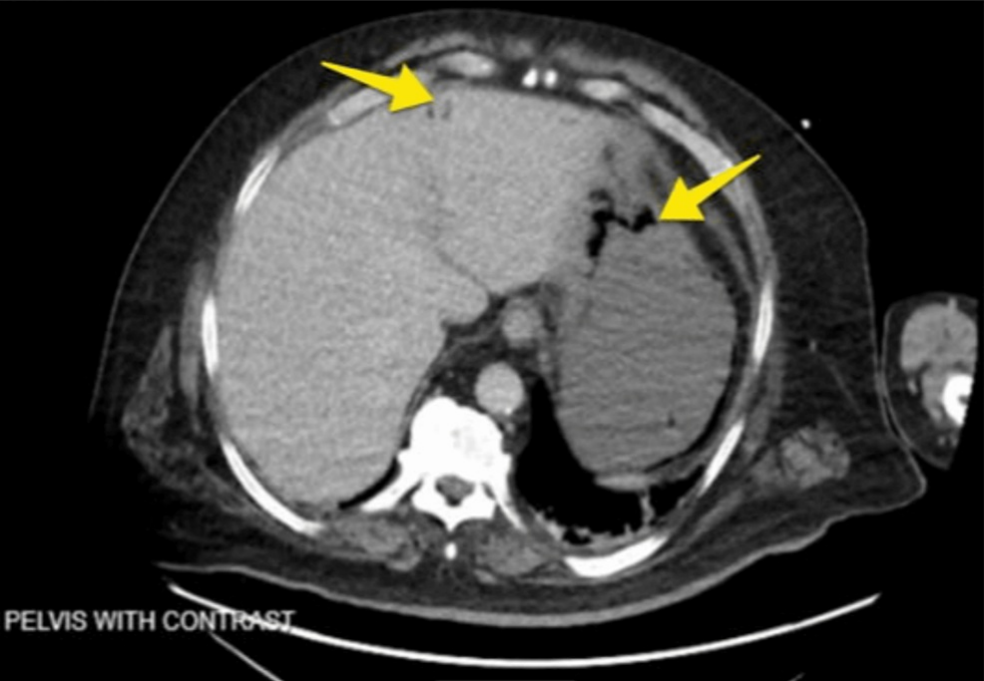
Axial view of CT scan of abdomen and pelvis with contrast showing gastric pneumatosis (right arrow) with portal venous gas (left arrow)

In ED, the patient was found to have elevated WBCs of 22.5 (4.5-11*10^3/uL) and lactic acid of 6.2 (0.5-2.0 mmol/L), as mentioned in Table 1. The patient was given intravenous fluids and broad-spectrum antibiotics. An abdominal CT scan was done for further evaluation of the pain, and it showed gastric pneumatosis with portal venous air (Figure 2). Surgery was consulted, and they initially recommended no emergent intervention, and a Nasogastric tube was placed to aid in stomach decompression. Gastroenterology was consulted for further management.

A repeat CT scan of the abdomen and pelvis was done after nasogastric tube decompression, which showed the persistence of the pneumatosis, and the patient failed to improve. The gastroenterology service was concerned about gastric perforation and referred endoscopy to the surgical service. The patient was taken to the operating room for an urgent laparoscopy and endoscopy for concerns of persistent gastric wall ischemia of unknown etiology, as the patient never had a previous endoluminal evaluation. A preoperative diagnosis of gastric ischemia was made. The patient underwent a diagnostic laparoscopy, esophagogastroduodenoscopy (EGD), exploratory laparotomy, partial gastrectomy, and drainage of the subphrenic abscess. The patient's postoperative course was complicated by respiratory distress requiring intubation for a brief period. The patient was eventually extubated. The patient tolerated the procedure well. The patient had a CT with oral contrast on postoperative day seven that showed no extravasation. The patient was started on a liquid diet which he tolerated well, and was discharged to rehab after having normal bowel movements. 

## Discussion

Pneumatosis intestinalis (PI) is the presence of gas and free air in the extraluminal space of the intestines [1]. The findings can be further categorized into benign pneumatosis intestinalis and life-threatening pneumatosis intestinalis [2]. The rare finding was first discovered by Du Vernoy during an autopsy specimen in the 1730s and later named pneumatosis cystoidies intestinalis (PCI) in 1825 [3]. The finding of PI can be considered primary or secondary. The primary subtype is a benign collection of gas that creates a cystic pattern with an incidence of 15%. The secondary subtype has an incidence of 85% and results from a pathologic condition resulting in a more linear gaseous pattern [1,4], which is seen in our cases. There are numerous different etiologies that make up the secondary causes of PI. The conditions associated with secondary PI have previously been categorized by Kahlil et al. into, but not limited to, causes mentioned in Table 2 [5]. It is important for clinicians to keep in mind the diverse etiology of the disease when working up pneumatosis intestinalis. 

**Table 2:** Common diseases and conditions associated with pneumatosis intestinalis COPD - chronic obstructive pulmonary disease, IBD - irritable bowel syndrome, PEEP - positive end expiratory pressure

Type	Diseases and conditions
Pulmonary	Asthma, COPD, emphysema, bronchitis, pulmonary fibrosis, cystic fibrosis
Gastrointestinal	IBD, diverticulitis, colitis, enteritis, intestinal obstruction, peptic ulcer, appendicitis, carcinoma
Autoimmune/systemic	Lupus, polymyositis, dermatomyositis, polyarteritis nodosa, scleroderma, celiac sprue
Infectious	HIV/AIDS, viruses, Candida albican, Mycobacterium tuberculosis
Iatrogenic	Endoscopy, enteric tube placement, PEEP ventilation
Drug-induced	Corticosteroids, lactulose, sorbitol, glucosidase inhibitors
Vascular	Mesenteric vascular disease, intestinal infarct, organ transplantation
Idiopathic (primary)	

The exact pathogenesis of the disease is not fully understood and is likely multifactorial. The three current theories of pathogenesis can be divided into mechanical, pulmonary, and bacterial [1,5]. The mechanical theory believes increased intraluminal pressure can lead to mucosal damage or iatrogenic trauma which allows gas into the extraluminal space which can then be propagated by peristalsis [1,5]. The pulmonary theory endorses that lung diseases such as chronic obstructive lung disease, asthma, or interstitial pneumonia can lead to alveolar rupture allowing gas to enter the mesenteric vascular by propagating from the mediastinum to the retroperitoneum and mesentery [1,5]. The bacterial theory believes that gas-producing bacteria can invade the intraluminal compartments [1,5]. With numerous possible etiologies and proposed pathogenesis, the radiographic finding of pneumatosis intestinalis can be difficult to treat. 

A retrospective study by Morris et al. showed the location of pneumatosis incidence to be 46% large colon, 27% small bowel, 5% stomach, and 7% both small and large bowel [6]. This study also showed a 43% mortality rate when the additional finding of portal venous gas is present. Our patient in case one presented with the rare radiographic finding of pneumatosis of both the stomach and duodenum along with portal venous gas (Figure 1). Case two imaging (Figure 2) showed the presence of gastric pneumatosis with portal venous gas. PI can be associated with pneumoperitoneum and portal venous gas, which carries a mortality rate as high as 75% [7]. We believe the radiographic findings in our patient in case one is from secondary pneumatosis intestinalis of the small bowel and stomach with the associated sinister finding of portal venous gas caused by small bowel obstruction. The treatment of secondary pneumatosis often depends on the etiology. Another difficult decision is whether or not the patient is in need of surgery. One article by Khalil et al. reported that surgery should be considered when there are concomitant CT scan findings of mesenteric ischemia, bowel obstruction, bowel perforation, or portal venous gas [5]. If these findings are not present, clinicians should perform a thorough investigation for critical labs, clinical presentation, and physical exam findings to further evaluate the need for surgery. 

In our second case, the patient was found to have gastric pneumatosis with portal venous gas. The stomach is the least common site of presentation, one retrospective study identified only 18 cases during a period of 15 years [8]. Gastric pneumatosis can be caused by mechanical force, pulmonary disease, bacterial infection, and ischemic condition, which often act concurrently [9]. In our second case, the causative etiology was unclear even after undergoing diagnostic laparoscopy, EGD, and exploratory laparotomy. More liberal use of CT scanning now a day has significantly increased the rate of detecting PI [10]. As seen in our second case, an early CT scan led to diagnosis in a timely manner. There are few reported cases of conservatively treating PI with good outcomes. Especially in the case of ischemic etiology with a resolution of PI after a few days without requiring surgical intervention [11]. But in case two, despite initial conservative management, serial CT scans showed persistence of PI, with the patient ultimately requiring surgical intervention. 

There is no standard treatment for this finding; most of the reported cases are treated conservatively [12]. In stable patients, resolution of emphysema within 72 hours has been reported with the use of nasogastric tubes for decompression [13]. A retrospective study by Morris et al. included 97 patients with intestinalis pneumatosis and reported a mortality of 16% in the group who received surgical treatment contrasting with a decreased mortality of 6% for the group who received conservative management [6]. The prognosis of gastric emphysema is usually benign with a complete resolution even without specific treatment [12].

The awareness of the numerous etiologies and subtypes of pneumatosis intestinalis would not only help guide treatment but may prevent morbidity and mortality. More studies are needed to standardize a treatment plan to address the increasing prevalence of pneumatosis intestinalis. 

## Conclusions

Pneumatosis intestinalis can occur from a wide range of etiologies, and clinical courses can vary from benign to life-threatening. Being able to recognize the radiographic findings among patients and the clinical significance can greatly affect morbidity and mortality. We report a case series of pneumatosis intestinalis associated with portal venous gas with different timing of surgical intervention. More research is needed to increase the awareness of pneumatosis intestinalis and formulate a standardized treatment plan to aid the decision of urgent surgical intervention versus observation.
